# Adult-Onset Neurological Deterioration in Dravet Syndrome Associated With a Novel SCN1A Missense Variant (p.Gly1371Asp): A Case Report

**DOI:** 10.7759/cureus.93228

**Published:** 2025-09-25

**Authors:** Koji Yokoyama, Sayaka Miyazaki, Kei Murayama, Ayumi Matsumoto, Kazuhiro Muramatsu

**Affiliations:** 1 Department of Pediatrics, Japanese Red Cross Wakayama Medical Center, Wakayama, JPN; 2 Diagnostics and Therapeutics of Intractable Diseases, Intractable Disease Research Center, Graduate School of Medicine, Juntendo University, Tokyo, JPN; 3 Division of Cardiovascular and Genetic Research, Department of Pediatrics, Jichi Medical University, Tochigi, JPN; 4 Department of Pediatrics, Jichi Medical University, Tochigi, JPN

**Keywords:** adult-onset neurological deterioration, bilateral basal ganglia calcification, dravet syndrome, mitochondrial dysfunction, scn1a mutation

## Abstract

We report the case of a 26-year-old woman with genetically confirmed Dravet syndrome (DS) who experienced atypical neurological deterioration in early adulthood, despite a previously stable clinical course. Her early history met the classical DS criteria, with the onset of febrile and afebrile seizures in infancy, followed by myoclonic, focal, and absence seizures, as well as developmental delay. Beginning at age 20, however, she developed new symptoms, including progressive tremor, postural instability, frequent gelastic-like seizures, and dysphagia severe enough to require temporary nasogastric feeding. Neuroimaging revealed basal ganglia and thalamic calcifications that had not been present in childhood. Mitochondrial evaluation demonstrated a mild reduction in oxygen consumption, despite normal enzyme activity. Genetic analysis, performed for the first time in adulthood, identified a previously unreported de novo missense variant in *SCN1A* (c.4112G>A, p.Gly1371Asp). This case highlights the potential for adult-onset phenotypic progression in *SCN1A*-related epilepsy and may broaden the clinical spectrum of DS, underscoring the importance of ongoing surveillance during adulthood, particularly in patients with rare or novel *SCN1A* missense variant (p.Gly1371Asp).

## Introduction

Dravet syndrome (DS) is a rare but severe developmental and epileptic encephalopathy, with an estimated incidence of 1 in 15,000-40,000 live births [1,2]. Most cases are caused by haploinsufficiency of the *SCN1A *gene, which encodes the neuronal sodium channel Nav1.1. DS is typically characterized by fever sensitivity, with seizures beginning during the first year of life, often triggered by febrile episodes, and evolving into multiple seizure types, including myoclonic, focal, and atypical absence seizures [3,4]. Developmental delay and cognitive impairment usually become evident during early childhood. Although profound impairment is common, the clinical course often stabilizes in adolescence, with seizure frequency decreasing and major neurological deterioration being uncommon in adulthood [2,5]. Adult-onset neurological deterioration, particularly involving motor, bulbar, or extrapyramidal symptoms, is rare and often suggests alternative or comorbid pathologies such as autoimmune encephalitis or mitochondrial disorders [6,7].

This report documents atypical neurological deterioration in adulthood associated with a novel *SCN1A* missense variant (p.Gly1371Asp), thereby broadening the recognized phenotypic spectrum of DS and highlighting the need for continued surveillance beyond adolescence. Brain imaging revealed new calcifications in the basal ganglia and thalamus, while metabolic evaluation demonstrated a mild reduction in mitochondrial oxygen consumption. Genetic reanalysis identified a de novo missense variant of *SCN1A* (c.4112G>A, p.Gly1371Asp), which has not been previously described in the scientific literature. This case illustrates the potential for adult-onset phenotypic progression in *SCN1A*-related epilepsy and expands the spectrum of late-onset manifestations associated with DS.

## Case presentation

The patient was a 26-year-old female who had been followed at our center since infancy for recurrent febrile and afebrile seizures, later recognized as consistent with DS. She first presented in April 2010 at age 11 years and seven months and was re-referred in December 2016 at age 21 due to progressive neurological decline. She was delivered at 36 weeks of gestation to non-consanguineous, healthy parents after an uneventful pregnancy, exhibiting normal psychomotor development during early infancy. There was no family history of epilepsy or developmental disorders.

Seizures began on the third day of life, initially presenting as short-duration focal motor seizures with alternating laterality, occurring up to 30 times per day. At eight months of age, she experienced her first prolonged febrile seizure following seasonal influenza vaccination, which required hospital admission and acute management with intravenous diazepam. At that time, she was otherwise in good health without preceding illness or fever. She recovered without acute neurological sequelae, and a rescue protocol with rectal diazepam (diazepam suppository) was prescribed for future prolonged seizures.

From nine months of age, she developed recurrent febrile and afebrile seizures, typically occurring about once or more per month. Between 18 and 20 months of age, she exhibited myoclonic seizures, focal dyscognitive seizures with cyanosis, and atypical absence seizures. During this period, multiple oral anti-seizure medications (ASMs; more than five agents in total) were trialed under the supervision of pediatric neurologists, but seizure control remained inadequate, and frequent seizures persisted. Developmental delay gradually became evident during early childhood.

**Figure 1:**
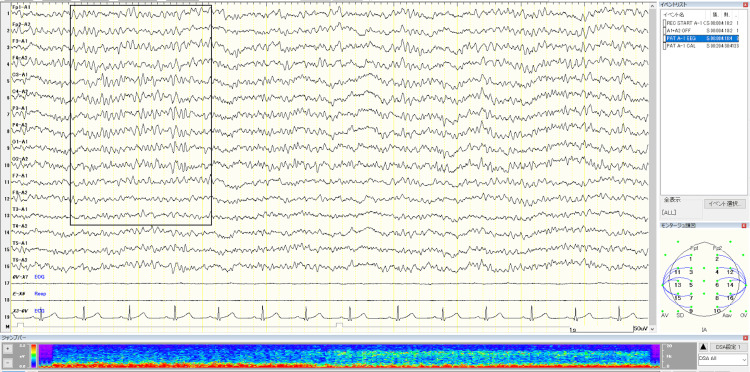
Interictal EEG at age 11 years and seven months (April 2010) Drug-induced sleep EEG demonstrating 12-14 Hz spindles (highlighted by black boxes) and abundant fast activity, without abnormal paroxysms or interictal epileptiform discharges. The boxes indicate representative normal sleep spindles in this tracing.

**Figure 2:**
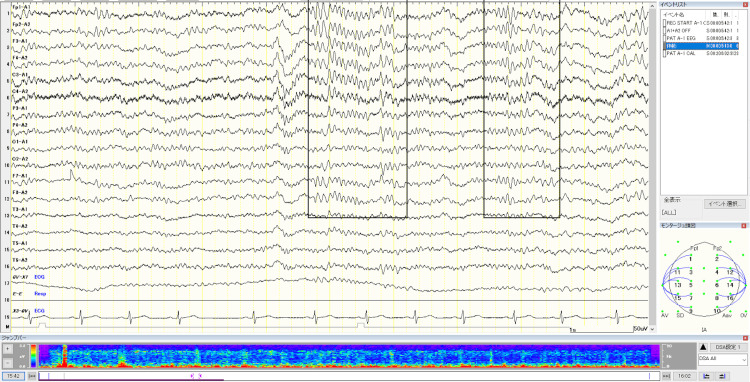
Interictal EEG at age 18 years and two months (December 2016) Sleep stage 1-2 EEG showing a hump 1 pattern and spindle activity (highlighted by black boxes), without abnormal paroxysms or interictal epileptiform discharges.

Despite frequent seizures, there were no abnormalities on the initial interictal EEG; however, multifocal and generalized spike-wave discharges emerged after 20 months of age. Earlier infant EEG tracings were unavailable. Representative later interictal EEGs are shown in Figure 1 and Figure 2.

**Figure 3:**
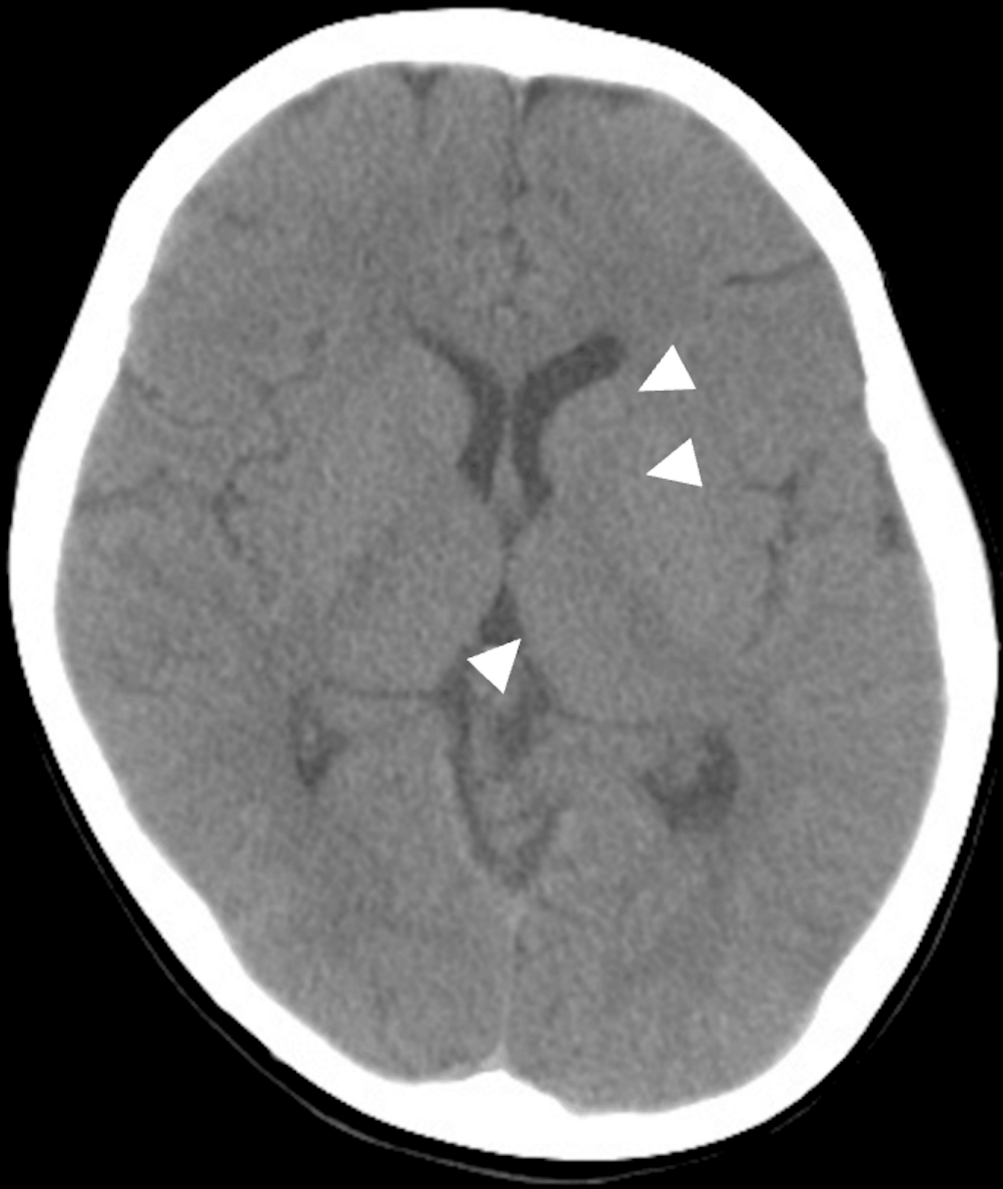
Non-contrast head CT at age 6 A non-contrast head CT shows no calcifications or structural abnormalities. White arrowheads indicate the basal ganglia and thalamus as regions of interest, where no abnormalities are present at this age. This normal scan serves as a baseline for comparison with later imaging.

Neuroimaging in early childhood was limited to head CT, which revealed no calcifications or structural abnormalities at age 6 (Figure 3).

She received multiple ASMs, including carbamazepine, phenytoin, zonisamide, clonazepam, ethosuximide, valproate, clobazam, acetazolamide, and potassium bromide; however, seizure control was not sustained. Developmental delay became apparent in early childhood, with an eventual diagnosis of DS based on clinical criteria consistent with those proposed by a North American consensus panel [1], including seizure onset before one year of age, multiple seizure types (febrile and afebrile generalized tonic-clonic, myoclonic, and focal seizures), slowing of cognitive and psychomotor development after seizure onset, and resistance to multiple ASMs.

Around 18 years of age, seizures gradually became more frequent. After age 20, however, the patient began to experience novel symptoms, including progressive tremor, leftward leaning posture, episodes of falling, and worsening dysphagia, which necessitated temporary placement of a nasogastric tube for enteral feeding. A representative video of gait training is provided (Video 1), which demonstrates her inability to ambulate independently, along with the emergence of significant motor dysfunction. Notably, she exhibited frequent laughter-like episodes resembling gelastic seizures, an atypical semiology for adult patients with DS, in whom seizure types typically stabilized and neurodevelopmental function became fixed.

**Video 1:** Assisted gait training of the 20-year-old patient with DS The video demonstrates the patient undergoing assisted gait training with full posterior support from a physical therapist. The patient is unable to walk independently, showing significant motor dysfunction and postural instability, which emerged in early adulthood, an atypical manifestation in the clinical course of DS. DS, Dravet syndrome

**Figure 4:**
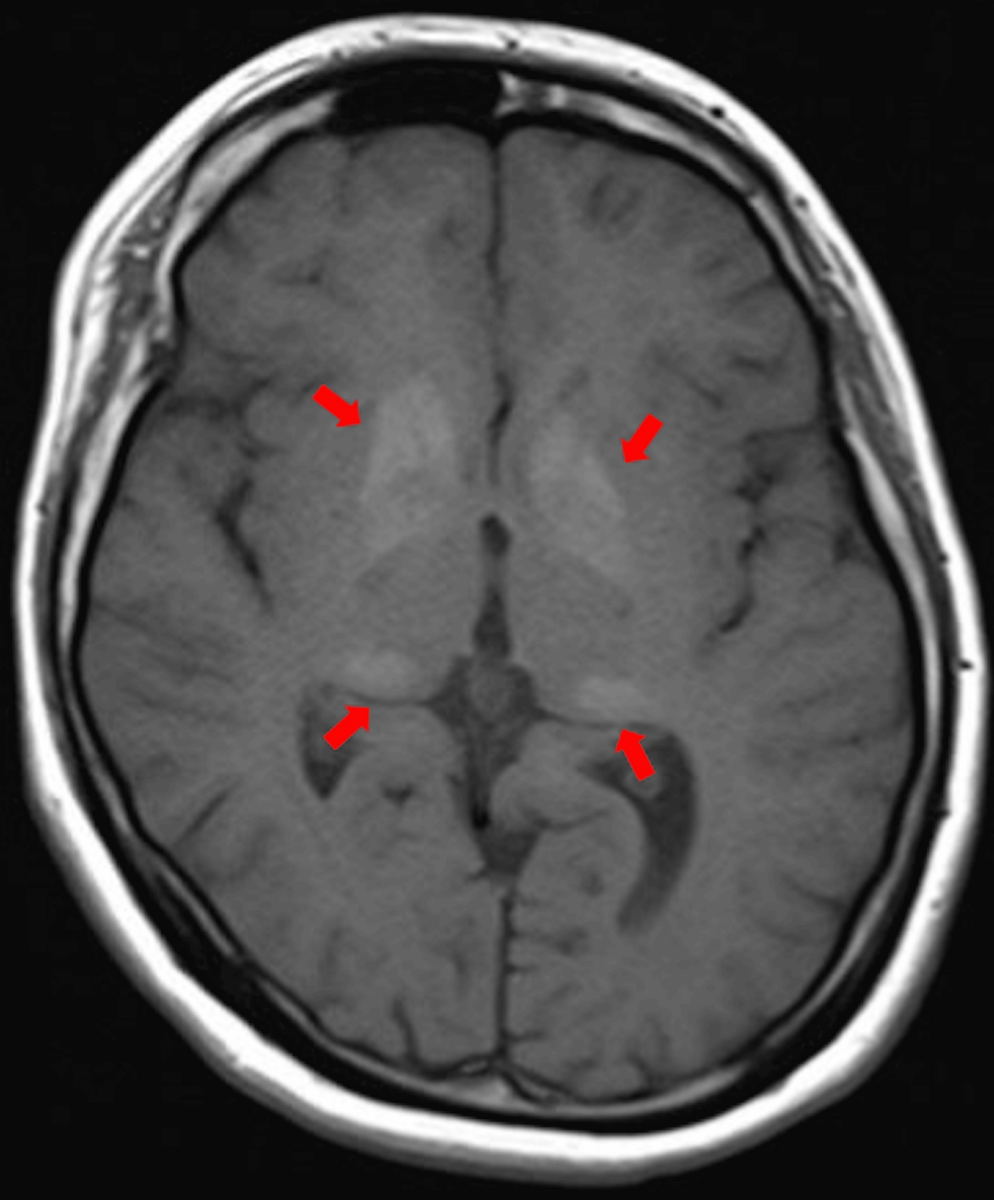
Brain MRI (T1-weighted imaging) at age 21 T1-weighted brain MRI shows faint hyperintensities in the basal ganglia (red arrows), which were not present on childhood imaging. These MRI changes correspond to the calcifications demonstrated on non-contrast head CT at the same age.

This unusual adult-onset deterioration prompted extensive reevaluation. A brain MRI in December 2016, at age 21, revealed faint T1-weighted hyperintensities in the bilateral globus pallidus, thalamus, and caudate nuclei (Figure 4). 

**Figure 5:**
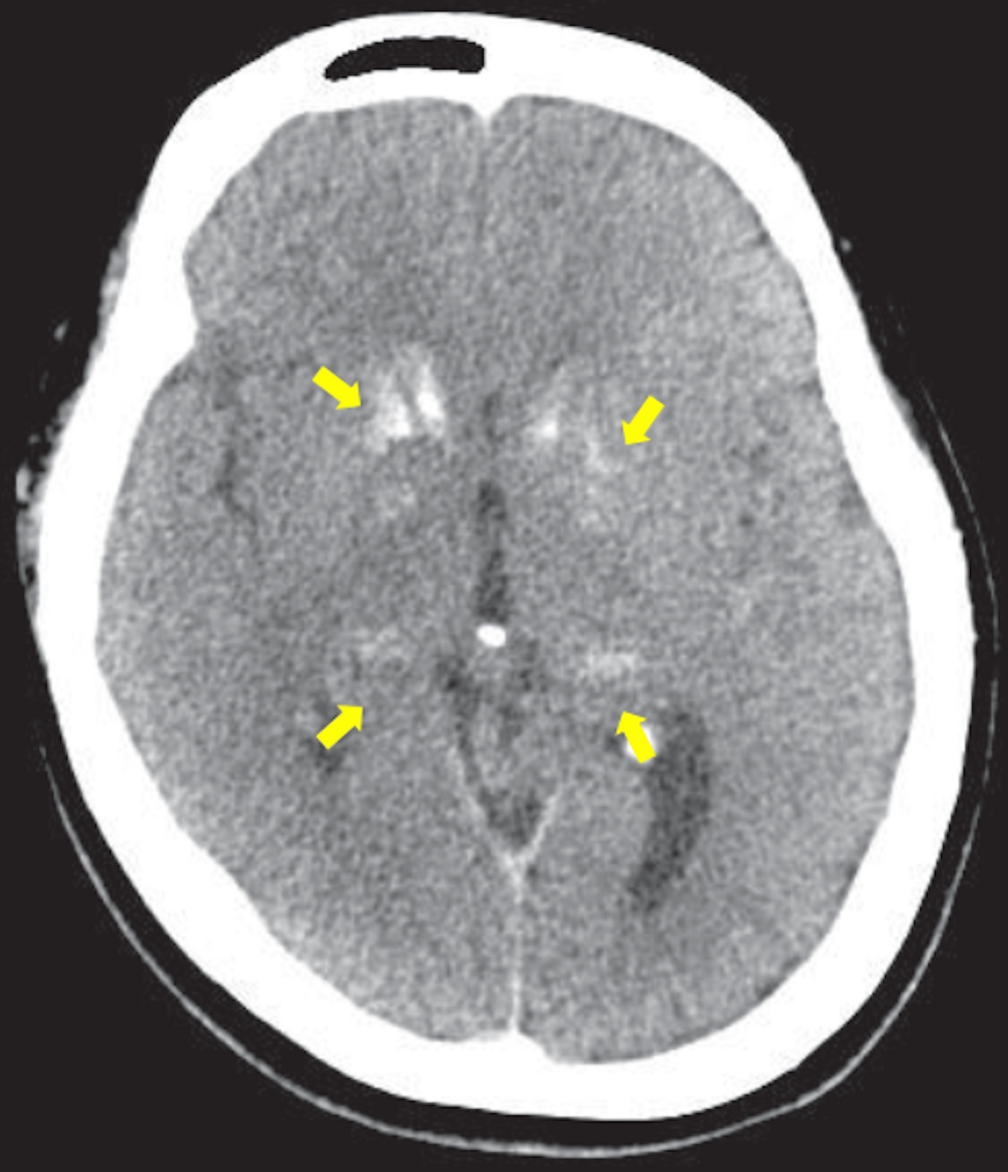
Non-contrast head CT at age 21 A non-contrast head CT reveals new calcifications (yellow arrows) located in the basal ganglia and thalamus, corresponding to the hyperintense regions observed on MRI. The interval appearance of calcifications underscores the progressive nature of structural changes, which were not present in childhood imaging.

A subsequent non-contrast head CT scan in December 2016 demonstrated calcification of the basal ganglia and thalamus (Figure 5), which had not been present on earlier images. CSF analysis at age 21 years is summarized in Table 1; all values were within normal limits, and the EEG showed no new epileptiform activity. Otolaryngology and ophthalmology evaluations revealed no structural abnormalities, although swallowing dysfunction was confirmed.

**Table 1:** CSF analysis at age 21 years CSF examination demonstrated normal findings, including a white blood cell count of 1/µL (mononuclear cells only), normal protein, glucose, lactate, and pyruvate levels, and a negative acid-fast bacilli culture. All parameters were within normal limits for the testing laboratory.

Parameter	Result	Reference range
White blood cells (/µL)	1	0-5
Mononuclear cells (/µL)	1	-
Polymorphonuclear cells (/µL)	0	-
Red blood cells (/µL)	0	0
Protein (mg/dL)	41	15-45
Glucose (mg/dL)	56	50-80
Lactate (mg/dL)	11.5	9-16
Pyruvate (mg/dL)	0.75	0.3-1.0
Acid-fast bacilli culture	No growth	Negative

Given the calcification of the bilateral basal ganglia identified on imaging, differential diagnoses such as metabolic disorders, idiopathic basal ganglia calcification (Fahr’s disease), and STING-associated vasculopathy with onset in infancy (SAVI) were considered. Laboratory tests were performed to evaluate iron metabolism, calcium-phosphate balance, and *STING1 *mutations associated with SAVI; all results were negative, effectively ruling out these conditions.

In addition, based on the patient’s clinical presentation, autoimmune encephalitis, particularly anti-N-methyl-D-aspartate receptor encephalitis, was suspected, and corresponding antibody testing was performed, which yielded negative results. Mitochondrial respiratory chain enzyme assays showed normal enzyme activity but mildly reduced oxygen consumption (41-44% of controls), consistent with prior reports of mitochondrial dysfunction in patients with DS [8]. Given this atypical clinical course, genetic testing of the *SCN1A* gene was performed for the first time and identified a heterozygous missense variant, c.4112G>A (p.Gly1371Asp). Parental testing confirmed that neither parent harbored this variant, supporting its classification as a de novo mutation.

According to ACMG/AMP guidelines, this variant was classified as “Likely Pathogenic,” supported by the following criteria: PM1 (location in a mutational hot spot and/or critical functional domain), PM2 (absence from control population databases), PM5 (novel missense change at a residue where another pathogenic missense change has been reported), PP2 (missense variant in a gene where missense changes are a common mechanism of disease), and PP3 (computational evidence supporting a deleterious effect). Moreover, the patient demonstrated mildly reduced oxygen consumption despite normal respiratory chain enzyme activities. Previous studies have reported similar findings of mitochondrial dysfunction in DS, raising the possibility that impaired bioenergetics could contribute to atypical neurological decline in adulthood.

Although this variant has been submitted to ClinVar (RCV001327577) as a variant of uncertain significance in the context of generalized epilepsy with febrile seizures plus (GEFS+) [9], it has not been previously reported in association with DS. It is absent from other population databases, affects a highly conserved residue, and is predicted to be pathogenic by multiple in silico tools. To the best of our knowledge, this is the first report of p.Gly1371Asp in a patient with DS, particularly in the context of progressive adult-onset neurological deterioration.

The patient was discharged from the hospital in 2020, at which time she was 21 years old. As of 2025, she is 26 years old and continues to live at home. Following discharge, she experienced daily gelastic seizures but no generalized tonic-clonic seizures (GTCs). She was able to ambulate indoors without assistance and remained in good spirits. Oral intake was partially resumed, and she could eat preferred foods, although mild pharyngeal stasis was noted during assisted feeding. Over the subsequent months, GTCs occurred once or twice per month, typically resolving spontaneously within a few minutes. Intercurrent febrile illnesses, including COVID-19, did not appear to affect seizure frequency. She is currently taking five ASMs. While daily gelastic seizures persist, she retains independent mobility and is able to eat a regular diet.

## Discussion

DS is a severe developmental and epileptic encephalopathy, typically caused by de novo mutations in the *SCN1A* gene, which encodes the alpha subunit of the voltage-gated sodium channel Nav1.1 [3,4]. The usual clinical course involves seizure onset during infancy, followed by cognitive decline and motor dysfunction in early childhood [1,10]. By adolescence or early adulthood, both seizures and neurodevelopmental deficits generally stabilize, with relatively little progression thereafter [11,12].

Our patient initially presented with the classical features of DS and followed the expected disease trajectory throughout childhood and adolescence. However, she later developed a constellation of novel neurological symptoms, including progressive tremor, postural instability, dysphagia, and laughter-like episodes resembling gelastic seizures, none of which are typical in adult DS patients with a stable course. This marked deterioration in adulthood prompted extensive investigation for alternative or coexisting diagnoses.

A brain MRI at age 21 revealed faint T1 hyperintensities in the bilateral globus pallidus, thalamus, and caudate nuclei. A non-contrast CT scan confirmed calcification in these same regions; these calcifications were absent on earlier childhood imaging. Taken together, these findings suggest a slowly progressive structural process, although one inconsistent with typical neurodegenerative, metabolic, or autoimmune etiologies. The presence of bilateral basal ganglia calcification raised the possibility of Fahr’s disease or other metabolic disorders, but targeted testing (including assessments of iron metabolism, calcium-phosphate balance, and mitochondrial markers) revealed no definitive abnormalities. Mitochondrial respiratory chain enzyme activity was normal, though oxygen consumption was mildly reduced (41-44% of controls), a finding previously reported in some DS patients [8].

Notably, the patient harbored a heterozygous de novo missense mutation in *SCN1A *(c.4112G>A, p.Gly1371Asp). The Gly1371 residue is highly conserved and lies within the S5 segment of domain III, a region crucial for voltage sensing and channel gating. While mutations in this domain have been implicated in typical DS presentations, the specific variant p.Gly1371Asp has not been previously described in the scientific literature and is extremely rare in public variant databases. It has been submitted to ClinVar as a variant of uncertain significance in the context of GEFS+ but not DS [9]. Multiple in silico tools predict it to be deleterious. According to ACMG/AMP guidelines, the variant is classified as “Likely Pathogenic,” supported by PM1, PM2, PM5, PP2, and PP3 criteria.

Progressive gait deterioration has been reported in adolescents with DS. A prospective analysis described crouched gait and musculoskeletal abnormalities in older patients, suggesting that progressive motor dysfunction can occur even without acute encephalopathy or structural abnormalities [13]. However, our case is unique in that neurological deterioration extended beyond gait disturbance to include features suggestive of bulbar involvement, such as progressive dysphagia requiring temporary nasogastric feeding, along with unusual seizure semiology characterized by frequent laughter-like episodes.

## Conclusions

This case illustrates an atypical pattern of adult-onset neurological decline in a patient with genetically confirmed DS associated with a novel *SCN1A* missense variant (p.Gly1371Asp), not previously described in the scientific literature. Unlike the typical disease course, which tends to stabilize during adolescence, this patient developed progressive motor symptoms, dysphagia requiring temporary enteral feeding, basal ganglia calcification, and mild impairment of mitochondrial respiration during early adulthood. While causality cannot be established from a single case, these findings suggest that this variant may have contributed to the patient’s unusual clinical course and expand the recognized phenotypic spectrum of *SCN1A*-related disorders. The observation of reduced oxygen consumption also raises the possibility of concomitant mitochondrial dysfunction, underscoring the importance of ongoing clinical monitoring into adulthood, particularly in individuals harboring rare or novel *SCN1A *missense variant (p.Gly1371Asp).

## References

[1] Wirrell EC, Laux L, Donner E (2017). Optimizing the diagnosis and management of Dravet syndrome: recommendations from a North American consensus panel. Pediatr Neurol.

[2] Genton P, Velizarova R, Dravet C (2011). Dravet syndrome: the long-term outcome. Epilepsia.

[3] Claes L, Del-Favero J, Ceulemans B, Lagae L, Van Broeckhoven C, De Jonghe P (2001). De novo mutations in the sodium-channel gene SCN1A cause severe myoclonic epilepsy of infancy. Am J Hum Genet.

[4] Brunklaus A, Brünger T, Feng T (2022). The gain of function SCN1A disorder spectrum: novel epilepsy phenotypes and therapeutic implications. Brain.

[5] Brunklaus A, Ellis R, Reavey E, Forbes GH, Zuberi SM (2012). Prognostic, clinical and demographic features in SCN1A mutation-positive Dravet syndrome. Brain.

[6] Selvarajah A, Zulfiqar-Ali Q, Marques P, Rong M, Andrade DM (2021). A systematic review of adults with Dravet syndrome. Seizure.

[7] Aljaafari D, Fasano A, Nascimento FA, Lang AE, Andrade DM (2017). Adult motor phenotype differentiates Dravet syndrome from Lennox-Gastaut syndrome and links SCN1A to early onset parkinsonian features. Epilepsia.

[8] Craig AK, de Menezes MS, Saneto RP (2012). Dravet syndrome: patients with co-morbid SCN1A gene mutations and mitochondrial electron transport chain defects. Seizure.

[9] (2025). SCN1A variant c.4112G>A (p.Gly1371Asp), RCV001327577. https://www.ncbi.nlm.nih.gov/clinvar/RCV001327577/.

[10] Brunklaus A, Zuberi SM (2014). Dravet syndrome—from epileptic encephalopathy to channelopathy. Epilepsia.

[11] Selvarajah A, Sabo A, Gorodetsky C (2025). Dravet syndrome: from neurodevelopmental to neurodegenerative disease?. Epilepsia.

[12] Nabbout R, Hyland K, Loftus R, Nortvedt C, Devinsky O (2024). Dravet syndrome seizure frequency and clustering: placebo-treated patients in clinical trials. Epilepsy Behav.

[13] Rodda JM, Scheffer IE, McMahon JM, Berkovic SF, Graham HK (2012). Progressive gait deterioration in adolescents with Dravet syndrome. Arch Neurol.

